# Supplementary figures and images for: Diagnostic accuracy of multiorgan point-of-care ultrasound compared with pulmonary computed tomographic angiogram in critically ill patients with suspected pulmonary embolism
Source: PLoS One. 2022 Oct 18;17(10):e0276202. doi: 10.1371/journal.pone.0276202 (PMC9578587; doi:10.1371/journal.pone.0276202)

## S1 File: STARD diagram

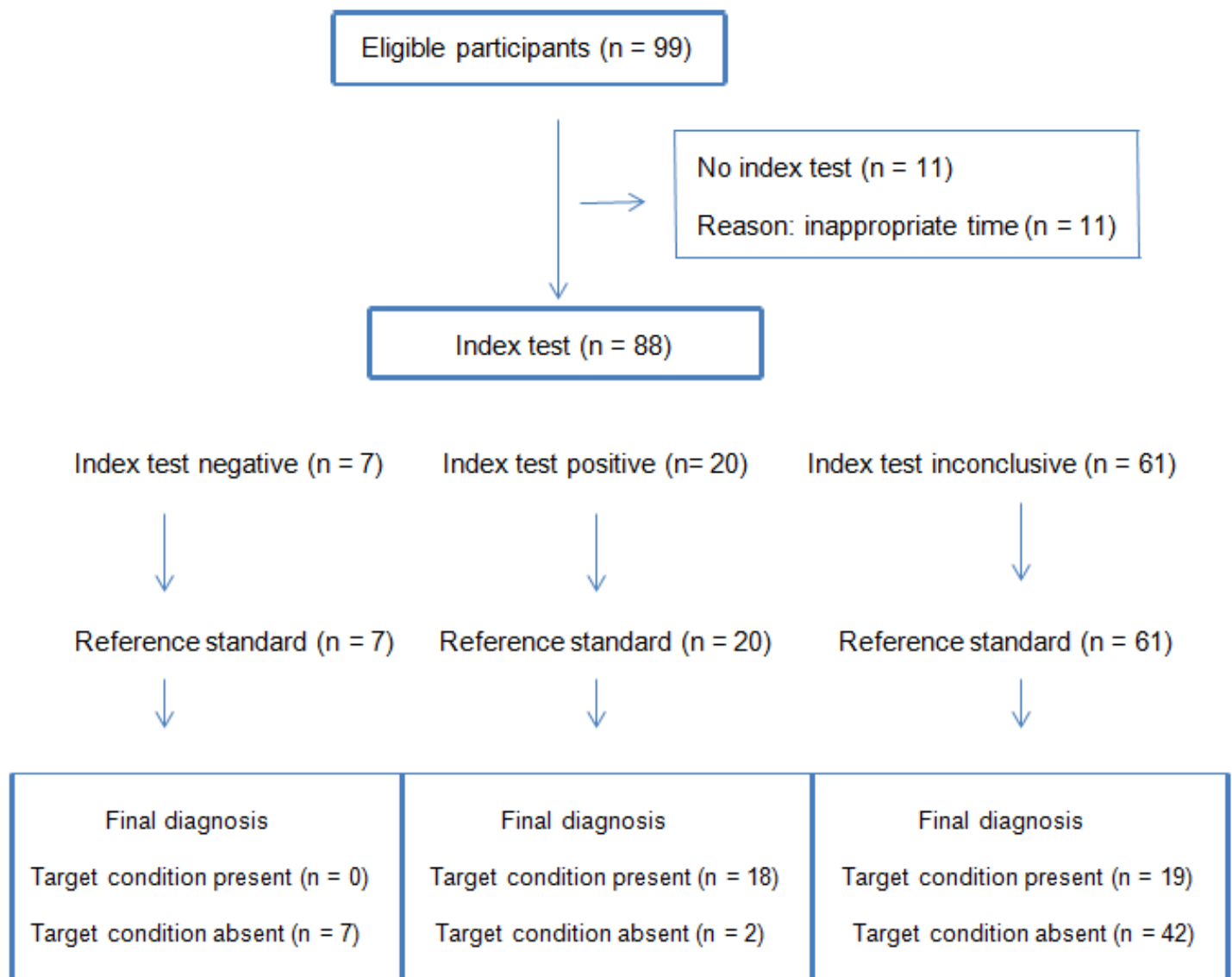

Supplement: S1 File — (PDF) [file pone.0276202.s001.pdf]
