# Supplementary material for: Diagnostic accuracy of multiorgan point-of-care ultrasound compared with pulmonary computed tomographic angiogram in critically ill patients with suspected pulmonary embolism
Source: PLoS One. 2022 Oct 18;17(10):e0276202. doi: 10.1371/journal.pone.0276202 (PMC9578587; doi:10.1371/journal.pone.0276202)
Supplement: S2 File — A: Troponin. B: NT-pro-BNP. (PDF) [file pone.0276202.s002.pdf]

S2 File: ROC curves demonstrate the performance of biochemical parameters in predicting pulmonary embolism.

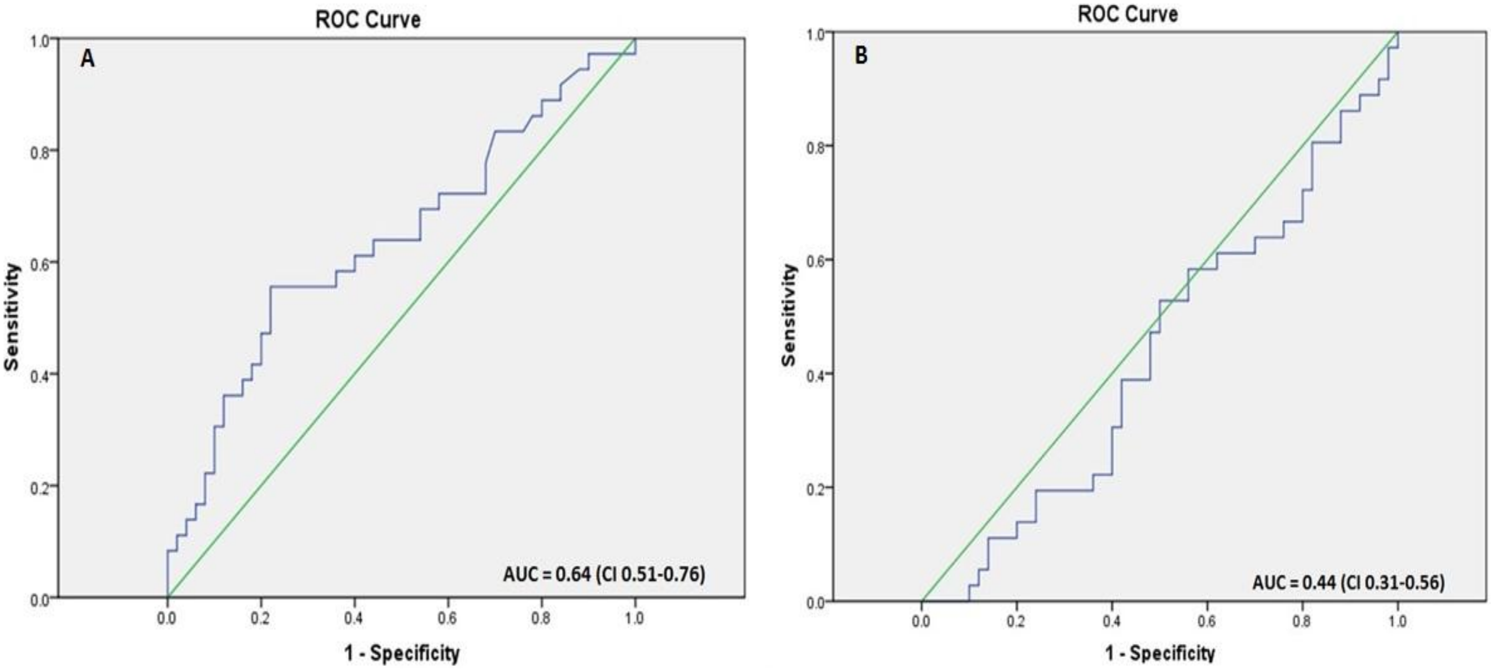

A. Troponin. B. NT-pro-BNP
