# Supplementary material for: Diagnostic accuracy of multiorgan point-of-care ultrasound compared with pulmonary computed tomographic angiogram in critically ill patients with suspected pulmonary embolism
Source: PLoS One. 2022 Oct 18;17(10):e0276202. doi: 10.1371/journal.pone.0276202 (PMC9578587; doi:10.1371/journal.pone.0276202)
Supplement: Attachment — Submitted filename: Response to Reviewers.docx [file pone.0276202.s004.docx]

Patricia Rezende do Prado
Academic Editor
PLOS ONE

**Ref: PONE-D-22-18103**

“Diagnostic accuracy of multiorgan point-of-care ultrasound compared with pulmonary computed tomographic angiogram in critically ill patients with suspected pulmonary embolism”

Thank you for reviewing the above referenced manuscript. The comments were very useful and the manuscript was amended accordingly. All changes made are highlighted in yellow in the revised version of the manuscript. Please see below the answers for all queries.

Do not hesitate to contact us if you require any further information.

Adriana Muradás Girardi

**Journal Requirements**

**Comment 1:** Please ensure that your manuscript meets PLOS ONE's style requirements, including those for file naming.

**Answer 1:** We thoroughly revised instruction to authors and made all arrangements to the files and figures to meet PLOS ONE's style requirements.

**Comment 2:** We note that the grant information you provided in the ‘Funding Information’ and ‘Financial Disclosure’ sections do not match. When you resubmit, please ensure that you provide the correct grant numbers for the awards you received for your study in the ‘Funding Information’ section.

**Answer 2:** As recommended, we provide the correct grant numbers for the awards in the ‘Funding Information’ section.

**Reviewer #1**

Thank you for your kind review. We incorporated your suggestions in the manuscript as follows:

**Comment 1:** Prediction scores as Wells and Geneva for pretest probability of PE validated in outpatient settings are occasionally used in the ICU. However, none of these clinical prediction rules have been validated in the ICU population. Therefore, more researches are needed to identify features that help to accurately identify patients with PE in this setting, and to develop a suitable prediction rule for critically ill patients with suspected PE. I think that in this study I do not see the relevance of considering these scores to analyze the usefulness of the multiorgan pocus. Please, comment about it.

**Answer 1:** This is an interesting point and we completely agree with the reviewer. The Wells and Geneva scores are used in an intensive care unit, but they are not validated in critically ill patients. There is no robust literature on the diagnostic investigation of PE in this population of patients. A previous study by our group (Ref 6, Wells and Geneva Scores Are Not Reliable Predictors of Pulmonary Embolism in Critically Ill Patients: A Retrospective Study) demonstrated that these scores were not good risk predictors for acute PE in critically ill patients, but the study was retrospective and we would like to confirm these findings in a prospective study. Then, we initially designed the study to include these scores in the analysis. However, our prospective results also showed low accuracy of these scores in critically ill patients with suspected pulmonary embolism (PE). Combining Wells and Genebra scores to mutiorgan ultrasound did not result I increased accuracy beyond the accuracy of multiorgan ultrasound. Therefore, we did not recommend to combine these scores with multiorgan ultrasound (please refer to Table 3 and Figure 1). We clarify this point in Discussion section (page 17, paragraph 1), as follows:

“Conversely, variables classically associated with PE, such as troponin and NT-proBNP [24], age [23], and the revised Geneva score [5], did not perform well in our population, in agreement with our previous retrospective results [6]. In this prospective study, combining the Wells and Geneva prediction scores with multiorgan scans did not increase diagnostic accuracy.”

**Comment 2:** In Table 2, define and explain better what it means “Alternative diagnosis on lung ultrasound”.

**Answer 2:** To better clarify what “Alternative diagnosis on lung ultrasound” means, we included a statement in Methods section (page 6, paragraph 2), as follows:

“The presence of one or more of the following lung abnormalities was considered an alternative diagnosis to PE on lung US: 1- the absence of pleural slip suggesting the presence of pneumothorax; 2- the presence of a hypoechoic pleural-based lesion suggesting consolidation or pulmonary atelectasis; 3- the presence of three or more B lines in an intercostal space in non-dependent lung areas suggesting alveolar-interstitial edema; 4- the presence of homogeneous anechoic area in a dependent lung area suggesting pleural effusion [15,16].”

**Comment 3:** Lung US performed by authors was based on diagnosis of acute respiratory failure method published on 2008. I would like to suggest that you make a more current and didactic classification shown in systematic review by Falster C et al.

**Answer 3:** Thank you for this important note. We used the paper by Falster et al. (Ref 16, please refer to References) to define the item "differential diagnosis" in pulmonary ultrasound. This point was not clearly stated in our article, but it is now better clarified as presented in the answer above (comment and answer 2) and in Methods section (page 6, paragraph 2). Unfortunately, some relevant points from the meta-analysis by Falster et al. were not incorporated into our POCUS analysis, for instance, the identification of pulmonary infarctions and McConnell’s sign. Despite the high specificity of these two findings, their recognition would require long training and senior expertise, but we chose a more easily reproducible approach.

**Reviewer #2**

This is an interesting study that looked into utilization of POCUS with clinical and lab values to diagnosed PE in comparison with CTPA.

**Comment 1:** "A noninvasive diagnostic strategy to help with timely decisions is important in critically ill patients" I think this statement should be modified as CTPA is already noninvasive, I would consider changing it.

**Answer 1:** We agree with the reviewer and changed the sentence as follows (page 4, paragraph 4):

“Therefore, alternative diagnostic strategies to help with timely decisions are important in critically ill patients.”

**Comment 2:** Definition of altered lung result on the basis of 3 or more altered fields may miss focal lung abnormalities like consolidation or atelectasis and this definition may under-diagnose alternative diagnoses to PE.

**Answer 2:** We agree with the reviewer. In all patients, we examined eight lung fields and collected two different variables in pulmonary ultrasound: 1- the presence of abnormal pulmonary findings; and 2- the presence of alternative pulmonary diagnosis (as better clarified in Methods section - page 6, paragraph 2). The absence of an alternative diagnosis on lung ultrasound (all lung fields considered) showed better performance and provided a more accurate information than the results of abnormal lung findings. Then we presented data on pulmonary ultrasound in a more objective way, referring only to the presence of alternative pulmonary diagnosis, which considered all lung fields examined.

The presence or absence of differential diagnosis on pulmonary ultrasound showed better performance in our analysis, being included in our final model of better diagnostic accuracy (please refer to Table 2). The definitions of alternative pulmonary diagnosis used in the article are the following: 1 - absence of pleural slip suggesting the presence of pneumothorax; 2 - presence of a hypoechoic pleural-based lesion suggesting consolidation or atelectasis; 3 - presence of three or more B lines in an intercostal space in no dependent lung areas suggesting alveolar-interstitial edema; 4 - presence of homogeneous anechoic area in dependent lung area suggesting effusion". Among these items, consolidations and atelectasis might be present in only one lung field, but due to its clinical relevance was considered an alternative pulmonary diagnosis. In this sense, we do not believe we have missed or under-diagnosed alternative lung diagnosis to PE.

**Comment 3:** The authors did not specify if RV dysfunction was acute or chronic as presence of previous RV dysfunction may confound the findings significantly. Also, I believe all patients with previous RV dysfunction should have been excluded from the study.

**Answer 3:** This is a very important point to clarify. We consider all patients to have acute RV dysfunctions. Few patients had previous echocardiography, none of them with right ventricular dysfunction or pulmonary hypertension. We assumed that patients without previous echocardiography would not have right ventricular dysfunction or pulmonary hypertension. We included a statement in Results, to better clarify the topic, as follows (page 11, paragraph 1 ):

“Of the 37 patients with positive findings for PE on CTA, 22 (60%) had RV dysfunction on POCUS TTE, with 63% sensitivity and 85% specificity. All patients were considered to have acute RV dysfunction.”

**Comment 4:** There was no mention of patients with history of previous PE or being treated for PE as these also may confound the findings.

**Answer 4:** This is a very important point in the evaluation of patients with suspected PE. Information on previous history of PE or VTD was analyzed in all patients, as this is one of the items used to calculate the Wells score, which was performed for all patients. In our sample, only two patients had previous PE or VTD and none of them were on treatment for PE or DTV in the last six months. This information was included in Results section (page 9, paragraph 1 of the results):

“Eighty-eight critically ill patients were included in the study. Two patients had a history of previous PE or DVT, but none of them were being treated for PE or DVT in the last six months.”

**Comment 5:** Were the images acquired by the fellows saved and reviewed by more experienced physician to concur with accuracy of ultrasound image acquisition given that ultrasound is operator dependent?

**Answer 5:** When we design the paper, we had a specific concern about this point. Critical care fellows were specifically trained for ultrasound image acquisition for the protocol for three months before to start the inclusion of patients. All images were saved and revised by a senior physician (author R.B.A). This point is clarified in Material and Methods (page 6, paragraph 1), as follows:

“POCUS examinations were performed independently by two critical care fellows with advanced training in POCUS who had undergone three months of training in image acquisition specifically for the study protocol. All images were saved and reviewed by a senior physician if necessary.”

**Comment 6:** The authors mentioned specificity of 100% in the abstract and then mentioned specificity of 96% in the results section, this should be corrected and clarified.

**Answer 6:** Thanks for pointing this error. The correct value for specificity is 96%. The information was corrected accordingly in Abstract (page 3, paragraph 1):

“Combining these three findings resulted in an area under the curve of 0.85 (95% CI, 0.77-0.94), with 50% sensitivity and 96% specificity.”

**Comment 7:** Given sensitivity of 50%, I think it is really difficult to state in the conclusion the diagnostic accuracy in high at this point. I would be more cautious with such a statement as the data presented does not fully support this conclusion.

**Answer 7:** We agree with the reviewer. The statement was changed as follows (page 18, paragraph 1, and in Abstract, page 3):

In Conclusion section:

“In conclusion, multiorgan POCUS combined with laboratory data has acceptable diagnostic accuracy for PE compared with CTA and might be helpful in the ICU setting, providing an alternative method when performing a CTA is too risky and reducing unnecessary tests when the probability of disease is too high.”

In Abstract section:

“Multiorgan POCUS combined with laboratory data has acceptable diagnostic accuracy for PE compared with CTA. The combined use of these methods might reduce CTA overuse in critically ill patients.”

**Reviewer #3**

**Comment 1:** I had a very difficult time determining what proportion of PEs were sub-segmental in nature.

**Answer 1:** As stated in Material and Methods (page 8, paragraph 1), thrombus location was defined according to the caliber of the affected vessel and classified as follows: main artery, lobar, segmental, or subsegmental. Scans were considered negative in the presence of adequate opacification of the pulmonary artery without filling defects, according to Moores et al. (included Ref 17, please refer to References). We had four patients with subsegmental PE (11% of patients with PE). This information was added to Results (page 9, paragraph 2), as follows:

“Of 88 patients, 37 (42%) had PE detected on pulmonary CTA examination. Of these, 12 patients (32%) had PE in the main artery, three (8%) in the lobar branch, 18 (49%) in the segmental branch, and four patients (11%) in the subsegmental branch.”

**Comment 2:** The paper also requires significant language editing.

**Answer 2:** Thanks for this important contribution to the final quality of the manuscript. A new round of language editing was performed. Paraphrasing that did not change the meaning of sentences is not highlighted in yellow. Please find below the English language editing certificate.

**Comment 3:** Abstract is lacking key information i.e. the sample size included in the study., in order to provide in the abstract an informative and balanced summary of what was done and what was found in the study, in accordance with STROBE statement.

**Answer 3:** Abstract was fully revised and all key information (including sample size) was amended accordingly with STROBE statement, in order to provide in the abstract an informative and balanced summary of what was done and what was found in the study. Please refer to Abstract section (page 2).
