# Supplementary material for: Diagnostic accuracy of multiorgan point-of-care ultrasound compared with pulmonary computed tomographic angiogram in critically ill patients with suspected pulmonary embolism
Source: PLoS One. 2022 Oct 18;17(10):e0276202. doi: 10.1371/journal.pone.0276202 (PMC9578587; doi:10.1371/journal.pone.0276202)
Supplement: Attachment — Submitted filename: Response to Reviewers.docx [file pone.0276202.s005.docx]

Patricia Rezende do Prado
Academic Editor
PLOS ONE

**Ref: PONE-D-22-18103**

“Diagnostic accuracy of multiorgan point-of-care ultrasound compared with pulmonary computed tomographic angiogram in critically ill patients with suspected pulmonary embolism”

Thank you for reviewing the above referenced manuscript. All changes made are highlighted in yellow in the revised version of the manuscript. Please see below the answers for all queries.

Adriana Muradás Girardi

**Journal Requirements**

**Comment 1:** Please review your reference list to ensure that it is complete and correct. If you have cited papers that have been retracted, please include the rationale for doing so in the manuscript text, or remove these references and replace them with relevant current references. Any changes to the reference list should be mentioned in the rebuttal letter that accompanies your revised manuscript. If you need to cite a retracted article, indicate the article’s retracted status in the References list and also include a citation and full reference for the retraction notice.

**Answer 1:** We thoroughly revised the reference list. In our last version of the manuscript, we added reference 17, which defines findings suggestive of PE on pulmonary computed tomographic angiogram. Also, we modified the position of reference 16 (previously reference 23), mentioned now in Materials and Methods and Discussion sections. In the current version of the manuscript, the references are all complete and correct. No reference cited that have been retracted. Reference list meets PLOS ONE style requirements.

**Reviewer #2**

I would like to thank the authors for addressing the comments on the manuscript.

**Comment 1:**  I think it would be appropriate that the authors acknowledge the lack of exclusion of previous RV dysfunction as weakness in the discussion section.

**Answer 1:** A statement was added to the Discussion section as follows (page 18, paragraph 1):

“Third, a formal exclusion of patients with previous RV dysfunction was not performed. However, no patients were suspected to have previous pulmonary hypertension based on previous echocardiography data and clinical data. Fourth, this is a single-center study.”

**Reviewer #3**

**Comment 1:** I am quite satisfied with the author's revisions of this manuscript. In particular, the attention paid to the inclusion of the STROBE statement elevates the project considerably.

**Answer 1:** We thank the reviewer for spending his time to improve your manuscript.
